# Permanent Draft Genome Sequences for Two Variants of *Frankia* sp. Strain CpI1, the First *Frankia* Strain Isolated from Root Nodules of *Comptonia peregrina*

**DOI:** 10.1128/genomeA.01588-15

**Published:** 2016-01-14

**Authors:** Rediet Oshone, Sheldon G. Hurst, Feseha Abebe-Akele, Stephen Simpson, Krystalynne Morris, W. Kelley Thomas, Louis S. Tisa

**Affiliations:** University of New Hampshire, Durham, New Hampshire, USA

## Abstract

*Frankia* stains CpI1-S and CpI1-P are members of *Frankia* lineage Ia that are able to reinfect plants of the *Betulaceae* and *Myricaceae* families. Here, we report two 7.6-Mbp draft genome sequences with 6,396 and 6,373 candidate protein-coding genes for CpI1-S and CpI1-P, respectively.

## GENOME ANNOUNCEMENT

Actinorhizal symbiosis is the association between an actinobacterium from the genus *Frankia* and a wide variety of dicotyledonous plants representing 8 different families of angiosperms, resulting in the formation of root nodule structure (1–3). The symbiosis allows these ecologically important pioneer plants to colonize harsh environments that are found worldwide (4). Molecular phylogenetic approaches have identified four major *Frankia* lineages (5–8). Cluster I contains two subclusters: one subcluster (cluster Ia) represents *Frankia* strains with the ability to infect a wider range of host plants, including members of the *Betulaceae* and *Myricaceae* families, and the other subcluster (cluster Ib) contains strains limited to *Casuarina* and *Allocasuarina* host plants. Members of cluster II infect host plants of the subfamily *Dryadoideae* (*Rosaceae*), *Coriariaceae*, and *Datiscaceae*, and of the genus *Ceanothus* (*Rhamnaceae*)*.* The members of cluster III are the most promiscuous and infect *Elaeagnaceae*, *Rhamnaceae*, *Myricaceae*, *Gymnostoma* (*Casuarinaceae*)*,* and occasionally *Alnus* species*.* The fourth *Frankia* lineage consists of the atypical strains, which are unable to reinfect actinorhizal host plants or form ineffective root nodule structures that are unable to fix nitrogen. The genomes for representatives from each cluster have been sequenced (9–24).

In 1978, the first *Frankia* isolate, CpI1, was obtained from root nodules of *Comptonia peregrina* and fulfilled Koch’s postulates (25). Following this initial success, many other strains from different host plants have been isolated, including the recent isolation of a member of cluster II (20). Two variants of *Frankia* sp. strain CpI1 were identified that had different carbon source requirements for growth. The *Frankia* sp. strain CpI1 succinate variant (CpI1-S) will use succinate and other dicarboxylic acids for growth, while the *Frankia* sp. strain CpI1 propionate variant (CpI1-P) will not (26). These two variants also show differences in their heavy metal tolerances and antibiotic resistance patterns (27, 28). *Frankia* sp. strains CpI1-S and CpI1-P were chosen for sequencing to provide more information on the differences between these two variants.

The draft genome sequences of *Frankia* sp. strains CpI1-S and CpI1-P were generated at the Hubbard Genome Center (University of New Hampshire, Durham, NH) using Illumina technology techniques (29). A standard Illumina shotgun library was constructed and sequenced using the Illumina HiSeq 2000 platform, which generated 10,867,000 reads (260-bp insert size) totaling 1,628.6 Mbp for CpI1-S and 20,978,588 reads (260-bp insert size) totaling 3,146.7 Mbp for CpI1-P. The Illumina sequence data were assembled using CLC Genomics Workbench (version 8.0.1) and AllPaths-LG (version r41043) (30). The final draft assembly for *Frankia* sp. CpI1-S consisted of 153 contigs containing a total sequence of 7,614,630 bp, with an *N*_50_ contig size of 99.4 kb and 206.3× coverage of the genome. For *Frankia* sp. CpI1-P, the final draft assembly consisted of 181 contigs containing 7,593,325 bp, with an N_50_ contig size of 98.1 kb and 287× coverage of the genome.

The assembled *Frankia* sp. CpI1-S and CpI1-P genomes were annotated via the Integrated Microbial Genomes (IMG) platform developed by the Joint Genome Institute, Walnut Creek, CA, USA (31, 32), and resulted in 6,396 and 6,373 candidate protein-coding genes for strains CpI1-S and CpI1-P, respectively.

### Nucleotide sequence accession numbers.

This whole-genome shotgun project has been deposited at DDBJ/EMBL/GenBank under the accession numbers JYFN00000000 (CpI1-S) and LJJX00000000 (CpI1-P). The versions described in this paper are versions JYFN01000000 and LJJX01000000, respectively.
